# Beneficial Role of Fruits, Their Juices, and Freeze-Dried Powders on Inflammatory Bowel Disease and Related Dysbiosis

**DOI:** 10.3390/plants11010004

**Published:** 2021-12-21

**Authors:** Maria Rosaria Perri, Carmen Romano, Mariangela Marrelli, Ludovica Zicarelli, Claudia-Crina Toma, Daniele Basta, Filomena Conforti, Giancarlo Statti

**Affiliations:** 1 Department of Pharmacy, Health and Nutritional Sciences, University of Calabria, 87036 Rende, CS, Italy; mariarosaria.perri@unical.it (M.R.P.); filomena.conforti@unical.it (F.C.); 2 SIACSA Società Italiana degli Analisti del Comportamento in campo Sperimentale ed Applicativo, 87100 Cosenza, RC, Italy; carmenromano78@yahoo.it; 3 Naturextralab S.R.L., 87040 Mendicino, CS, Italy; ludovica.zicarelli@gmail.com; 4 Pharmacognosy Department, Faculty of Pharmacy, Vasile Goldis Western University of Arad, 87 L. Rebreanu Str., 310045 Arad, Romania; claudiatoma2004@yahoo.com; 5 University Sport Center, University of Calabria, 87036 Rende, CS, Italy; nutrizione.danielebasta@gmail.com

**Keywords:** fruits, gut microbiota, inflammatory bowel disease, juices, lyophilized powder

## Abstract

Inflammatory bowel disease (IBD) is a group of complex chronic inflammatory conditions affecting the gastrointestinal tract. It is linked to a number of genetic and environmental factors able to perturb the immune-microbiome axis. Diet is the most investigated variable both for its role in the etiology of IBD and for its beneficial potential in the treatment of the symptoms. Dietary products may influence intestinal inflammation through different mechanisms of action, such as the modulation of inflammatory mediators, the alteration of gene expression, changes in gut permeability, and modifications in enteric flora composition. A consisting number of studies deal with the link between nutrition and microbial community, and particular attention is paid to plant-based foods. The effects of the dietary intake of different fruits have been investigated so far. This review aims to present the most recent studies concerning the beneficial potential of fruit consumption on human gut microbiota. Investigated plant species are described, and obtained results are presented and discussed in order to provide an overview of both in vitro and in vivo effects of fruits, their juices, and freeze-dried powders.

## 1. Introduction

Inflammatory bowel disease (IBD), mainly including ulcerative colitis (UC) and Chron’s disease (CD), represents a cluster of chronic, multifactorial, incurable pathologies with a low rate of mortality [1]. It is a permanent and disabling disease that occurs at a young age, with abdominal pain, diarrhea, anemia, and slimming as the most recognized symptoms. Until 2016, IBD syndrome seemed to be more prevalent in developed Western countries but, more recently, a significant increasing incidence has also been observed in Asia. The cost of living for a patient with IBD seems to be relatively high in many different countries: Europe has an estimated health system cost of €4.6–5.6 billion per year. Specifically, UC and CD are severe health problems diffused all over the world with an incidence rate of 12.7 and 24.3 per 100.000 person-year and a prevalence of 0.5% and 1%, respectively, in Europe [2]. For what concerns sex, data show an increased occurrence of CD in females and a major UC onset in males: male dominance is observed during childhood while a female majority is revealed starting from puberty. Generally, almost a year passes from the recognition of the first symptoms to the diagnosis of the disease itself; the course of this pathology is unpredictable. It depends on the medication and patient response to treatments. As the IBD pathophysiology has not been fully elucidated, it is treated as a manageable chronic condition that implicates a lifelong use of drugs [3,4,5]. It has a progressive and destructive course, leading to a series of complications such as stenoses, abscesses, fistulas, extraintestinal manifestation, colitis-associated neoplasia, and cancer: it is a chronic status, and its management requires several drugs such as corticosteroids, immunosuppressants, and anti-tumor necrosis factor-alpha(TNF-α) antibodies. Surgery is the last chance, and often, it can be correlated with complications. Drugs such as anti-TNF agents and vedolizumab have been recently introduced for IBD therapy, allowing to both reduce corticosteroid use and increase response and remission rate. Other drugs, such as azathioprine plus infliximab, act synergistically, are effective in suppressing immune cell activation and gut inflammation. MicroRNAs (miRNAs) in the intestinal tract cooperate to tissue homeostasis, intestinal cell differentiation, maintenance of the intestinal barrier function: research is aimed at using them as potential biomarkers able to make a diagnosis and to predict both the course of pathology and the response to treatment [2].

Recent studies have shown that almost 30–50% of IBD patients do not respond to anti-TNF therapy: as a consequence, the research currently focuses on nutraceutical compounds that could be useful in the treatment of IBD patients [6,7,8]. In fact, nutrition plays a double role both in IBD onset and management: it is involved in UC and CD pathology, and it has a central role in disease treatment. In children with IBD, for example, nutritional support is fundamental because it represents an alternative to pharmacological treatments. Epidemiological data indicate that an imbalance between lipids and fatty acids and a diet rich in animal proteins are predisposing factors to IBD disease, while the daily intake of vitamin D, fibers, vegetables, fruits, n-3 polyunsaturated fatty acids (n-3 PUFA), and n-9 polyunsaturated fatty acids (n-9 PUFA) can exert a protective role [9,10]. The “Western diet” (high carbohydrate/high fat/low fiber) can lead to severe dysbiosis. Gut microbiota dysbiosis, an alteration of the microbial community that consists of an imbalance between harmful and protective bacteria, has been widely described in IBD patients. On the contrary, the “Mediterranean diet” and the vegetarian one, rich in food with anti-inflammatory properties such as fruits, vegetables, and olive oil, seems to be able to prevent dysbiosis and, consequentially, IBD [11,12].

## 2. Inflammatory Bowel Disease (IBD)

IBD, mainly including UC and CD, represent a complex of heterogeneous, idiopathic, chronic conditions that result in an uncontrolled inflammation of the gastrointestinal (GI) tract [3].

The evolution of IBD can be stratified into four epidemiological stages: emergence, acceleration in incidence, compounding prevalence, and prevalence equilibrium. In this historical period, developing countries are in the emergence stage, industrialized ones are in acceleration in incidence stage, while Western regions are in the third stage. Actually, every region of the world can be located in one of the first three IBD epidemiological stages [13]. There are many factors that can affect IBD development: genetic, ethnic, and environmental ones. Smoking, infections, intestinal microbiota, and immune component are involved in the IBD pathogenesis. Moreover, anomalous permeability of the mucosal barrier, microflora composition, and immune system, together with oxidative stress, seem to lead to the pathology assessment [6,7]. Early life events (birth, breastfeeding) as well as other occurrences during life, such as exposition to air pollution or hypoxia associated with high altitude, can be considered potential risk factors for IBD onset [14]. Genetic analysis suggested that over 230 genes are involved in the predisposition of IBD, and all these genetic polymorphisms are strictly connected with the mucosal barrier function [15]. Although most of the reported IBD cases are due to polygenic susceptibility, a very low number of rare genetic diseases that produce IBD-like intestinal inflammation are described. Monogenic defects can cause very-early-onset inflammatory bowel disease (VEO-IBD) that appear before two years, while early-onset inflammatory bowel disease (EO-IBD), which can manifest itself before 5 years, is often reported as CD or CD-like pathology [2].

The CD onset has undergone a significant increase in industrialized countries in the last 50 years. Food intake is also considered an environmental risk factor in the IBD onset: evidence showed that the consumption of fruit and vegetables is correlated to a decreased risk of contracting CD, while the consumption of a diet rich in fat and sugar can lead to a predisposition of CD development. Moreover, it seems that medium-chain fatty acids can promote intestinal inflammation if compared to long-chain ones. In addition, psychological stress, appendectomy, and medications should not be underestimated in the frame of IBD environmental risk factors [16].

Currently, the exact etiology of IBD has not yet been understood, and it is not clear whether such gut microbiota alteration is a cause or a consequence of intestinal inflammation. According to the most reported hypothesis, IBD could result from an excessive immune response toward the altered gut microbiota or some microorganisms in a genetically prone host [17].

Novel treatment options targeting immune pathways have been introduced for the treatment of IBD in the last two decades. Even if anti-TNF agents still represent the first-line option for the treatment of moderate to severe UC and CD, a treatment failure is observed in a significant percentage of patients, which requires alternative agents. New and emerging therapies targeting immune pathways include anti-adhesion agents and anti-interleukin inhibitors. The first class of drugs, including natalizumab and vedolizumab, prevent lymphocyte infiltration by blocking the action of integrins, the adhesion molecules involved in the recruitment of inflammatory cells to intestinal lesions. Anti-interleukin inhibitors include ustekinumab and risankizumab [18,19].

Moreover, Janus kinase/signal transducer and activator of transcription (JAK/STAT) inhibitors, such as tofacitinib and filgotinib, are receiving much attention in the development of novel IBD therapies, besides the interest for the treatment of other inflammatory conditions [20,21].

Furthermore, sphingosine-1-phosphate receptor modulators constitute another new and promising approach for the treatment of a range of inflammatory disorders, including IBD [22].

## 3. Gut Microbiota

The gut is composed of trillions of microorganisms living symbiotically with the host [23]. About 1000 different bacterial species inhabit the intestine in a concentration of 100 trillion with a density of 9 × 10 ^13–14^, while the colon is populated by 160–500 different bacterial species [12]. All these microorganisms (commensal bacteria, viruses, fungi, protozoa, and archaea) compose the microbiota [24]. The microbiota genome is called the microbiome [25]. The fetal gut appears sterile during pregnancy, as its colonization occurs at birth. At the passage through the birth canal, in fact, the neonatal intestine came to be populated by anal and/or vaginal maternal flora containing *Bacteroidetes*, *Bifidobacterium*, *Prevotella,* and *Lactobacillus* spp. [12]. *Bacteroidetes* and *Firmicutes* are the most prevalent phyla in the human gastrointestinal tract, followed by *Actinobacteria*, *Proteobacteria,* and *Verrucomicrobia* [26].

A mutual symbiotic relationship with the human host exists: the gut offers a habitat rich in nutrients, while the microbiota offers protection against pathogenic organisms, and it is able to metabolize toxins and drugs. Under physiological conditions, microbiota represents a homeostatic organ involved in the maintenance of the intestinal mucosa integrity and in the synthesis of some vitamins, fermentation of complex polysaccharide polymers, and short-chain fatty acids [17]. Moreover, metabolites such as butyrate and lactic acids that provide anti-inflammatory, anti-tumorigenic, and anti-microbial properties in the host can be produced by microbiota [27,28]. A dysregulated gut microbiome composition causes a large spectrum of inflammatory diseases such as irritable bowel syndrome (IBS), IBD, and cancer [29].

Different studies showed that the gut microbiome is strictly connected to psychosis and behavior: the gastrointestinal tract and brain influence themselves reciprocally. Recent studies show that a malfunction in the gut-brain network is responsible for gut inflammation disorders, alteration in behavior, acute and chronic stress responses. Different pathways play a role in the communication between gut microbiota and the brain, such as vague nerve and enteric nervous system, but the exact mechanism through which bacteria of the GI tract can influence brain and behavior is still unknown. Neuromodulatory metabolites derived from microbiota are tryptophan precursors and metabolites, serotonin, GABA, and catecholamines. These products interact with host cells and synapses of the autonomic nervous system. The hypothesis of influence between enteric microbiota, brain, and behavior leads to the concept of the microbiota-gut-brain axis [30].

## 4. Dysbiosis and IBD

A compositional and functional alteration of the microbiota caused by both environmental and host-related factors is commonly called dysbiosis. This change in the gut microbiome setup can lead to beneficial, neutral, or dangerous consequences for the host. Loss of variety, loss of commensal, and blooming of pathobionts (commensal microbiota with the potentiality to cause pathology) are the main features of dysbiosis. The most recognized factors in the pathogenesis of dysbiosis are: nutrients, drugs, intestinal mucosa, and immunity [31].

The immune system seems to play a crucial role in establishing normal and dysbiotic microbiome. In fact, a healthy or dysbiotic microbiota can affect the host’s innate immune system through the composition of microbial cell components and metabolites [32].

Gut dysbiosis is often found in patients with CD, IBD, obesity, immunological defects, and abnormal behaviors in children [29,30,33,34].

For a long time, the gut microbiome was considered the key player in the pathogenesis of IBD; currently, there seems to be unanimous consent in the association of IBD with dysbiosis, defined as a decrease in gut microbiome variability that leads to an imbalance between commensal and potential pathogenic microorganisms. Clinical and experimental data suggest that dysbiosis is implicated in the IBD pathogenesis: the alteration in gut microbiota composition is translated in a damaged host immune system and lack of barrier integrity that leads to aberrant immune response and the onset of chronic inflammation [35].

Different studies demonstrated a link between gut microbiota and inflammatory diseases: both those affecting the gastrointestinal tract, such as CD and UC, and other diseases, such as multiple sclerosis and rheumatoid arthritis, have been taken into account [36]. Moreover, the role of gut microbiota was also highlighted in the pathogenesis of obesity [37] and diabetes [38].

*Enterobacteriaceae*, a large class of Gram-negative facultative bacteria, are commonly linked to IBD, as well as the depletion of the phyla *Firmicutes* and the increase in *Proteobacteria* [39].

Short-chain fatty acids (SCFAs) also play an important role, as they exert anti-inflammatory activity via binding to G-protein-coupled receptor 43 (GPR43), which is expressed in immune cells, including macrophages. SFCAs, such as acetate, propionate, and butyrate, are major products of the microbiota digestion of plant fiber polysaccharides in the human colon [40]. Different studies demonstrated a decreased level of fecal SCFAs in patients with IBD, as well as an increased level of the intermediate molecule lactate in patients affected by CD and UC [41].

Furthermore, an increased tryptophan metabolism has been identified in IBD. This essential amino acid can be metabolized by the gut microbiota into a range of indole metabolites [41].

The relationship between gut microbiota and inflammation has been deeply described by Wang and colleagues, who also discussed the important role of macrophages [39]. Intestinal macrophages are the first-line immune cells against the invasion of pathogens, and they also produce anti-inflammatory cytokines such as IL-10 and further regulate T-cell differentiation to prevent mucosal auto-inflammation. For these reasons, intestinal macrophages are important for maintaining intestinal homeostasis.

The hypothesis that gut microbiota can pave the way for inflammation in IBD sparked a large series of clinical approaches aimed at correct dysbiosis with dietary and/or microbial intervention thanks to the use of probiotics, antibiotics, enteral nutritional therapy (ENT), and fecal microbiota transplantation (FMT) [42].

## 5. Research Methodology

This search includes references published up until October 2021. The following academic search engines have been used: Google Scholar, Pubmed, and Scopus. The keywords “gut microbioma”, “inflammatory bowel disease”, and “fruit” have been searched in the databases combining descriptors by using the Boolean operators “and” or “+”. Only articles, reviews, and book chapters were searched, while other document types such as Meeting Abstract and Proceeding Paper were excluded. Both in vitro and in vivo studies have been taken into account.

## 6. Fruit Intake and Potential Benefits on Gut Microbiota

The beneficial role of fibers in a healthy diet pattern is well known in literature, including healthy aging promotion, overweight and obesity risk reduction, several cancers, and type 2 diabetes risk decrease [43]. Epidemiological studies have also demonstrated the inverse association between vegetable intake and cardiovascular diseases [44,45,46].

Whole fruits are known to be a suitable source of fibers, with low-moderate energy density levels. Moreover, fruits contain important micronutrients and phytochemicals, which may exert many positive health effects acting in synergy [47]. Dietary fibers are edible carbohydrate polymers with three or more monomeric units resistant to digestive enzymes in the gut and, as a consequence, not absorbed in the upper gastrointestinal tract. These polymers are present mainly in cereals, fruits, and vegetables [48]. It has been observed that dietary fibers from plant sources and plant phytochemicals may mediate their observed protective effects through their interaction with gut microbiota [49]. Fruit fibers are important for their prebiotic gut effects, including the growth of beneficial bacterial species and the promotion of healthy microbiome diversity and composition, mainly due to local fermentation in SCFAs [48,50,51]. In particular, pectin and polyphenols combination, typical of whole fruits, can influence the microbiome composition increasing the number of *Actinobacteria* and *Bacteroidetes* and decreasing the number of *Proteobacteria* and *Firmicutes* [52]. Evidence shows that fruit fiber intake, especially pectin, plays a pivotal role in correcting GI inflammation and promoting microbiome health, improving gut-brain axis communication [53].

Thumann and colleagues provided a very interesting overview of medicinal plants traditionally used in Europe for the treatment of gastrointestinal disorders and the potential interaction with human gut microbiota [54]. The beneficial role of many vegetables, such as pumpkin, spinach, broccoli, and beet, has been investigated as well [55].

The influence of different phytochemicals, such as flavonoids, phenolic acids, lignans, and terpenoids, in the prevention and treatment of inflammatory bowel disease [56,57,58] has also been previously reviewed. Particularly, different studies focused on the role of polyphenols in modulating gut microbiota and intestinal immune function and in inhibiting intestinal inflammation [49,52,59,60,61,62,63,64]. A two-way interaction has been observed between polyphenols and gut microbiota [60]. Once ingested, the bioavailability of polyphenols is low: low-molecular-weight compounds may be absorbed in the small intestine, while polymeric polyphenols such as condensed tannins reach the colon almost unchanged. About 90% of total polyphenols intake accumulate in the large intestinal lumen, where they are subjected to the activity of colonic microbiota [62], which allows the production of bioactive metabolites better absorbed than ingested phenolic compounds. These molecules, in turn, have been demonstrated to affect gut microbiota composition by increasing beneficial genera such as *Lactobacillus* and *Bifidobacterium* [59].

**Figure 1:**
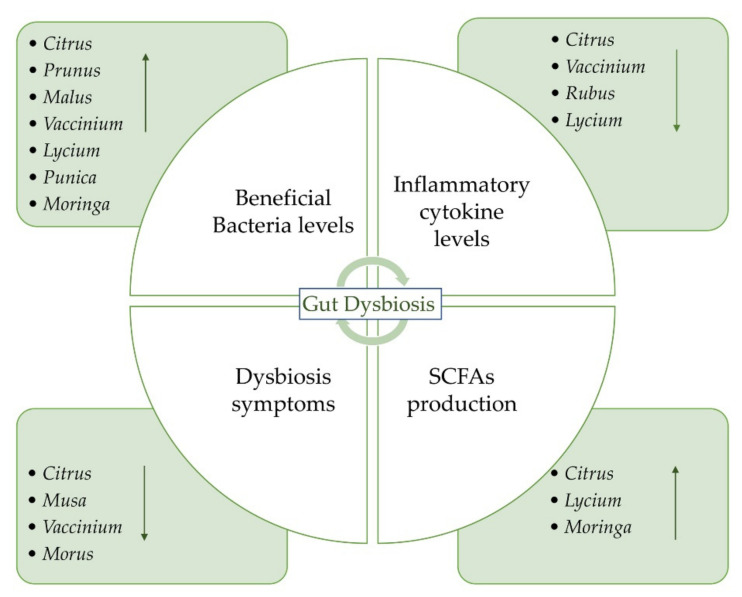
The gut microbiota dysbiosis process and potential role of fruits, their juices, and freeze-dried powders on IBD.

**Figure 2:**
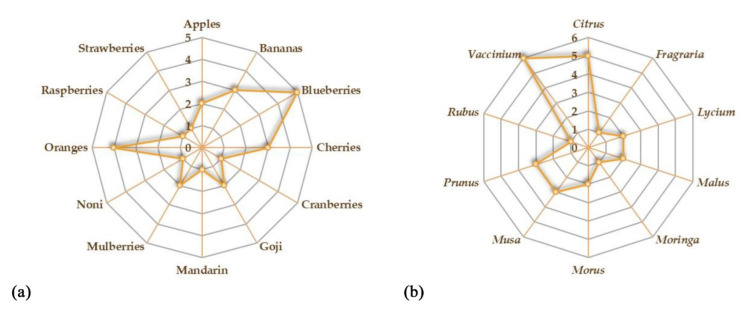
Kiviat diagrams visualizing the number of studies focusing on the beneficial effects of fruits (**a**) and plant genera (**b**) on gut microbiota.

The aim of this review is to provide an overview of the studies concerning the potential beneficial effects of fruits on gut microbiota and IBD (Figure 1). We reported the recent evidence about the potential role of some fruits that could be administered as such or as juice. The beneficial effects of freeze-dried powders from fruits have also been reported. Both in vitro and in vivo studies on the possible effects of the consumption of different species on gut microbiota have been taken into account. A summary of all the studies concerning the potential beneficial effects of fruit supplementation is provided in Table 1. Oranges and blueberries are the most investigated fruits for their potential beneficial properties on gut microbiota (Figure 2a). On the whole, *Vaccinium* is the most investigated genus, followed by *Citrus*, *Prunus,* and *Musa* genera (Figure 2b).

### 6.1. Orange and Mandarin (Citrus Genus)

Fruits belonging to the *Citrus* genus are among the most popular and consumed fruit types worldwide. They are rich in health-promoting compounds, mainly flavonoids, well known for a number of beneficial effects, such as antioxidant, anti-inflammatory, and anticancer activities [92].

**Figure 3:**
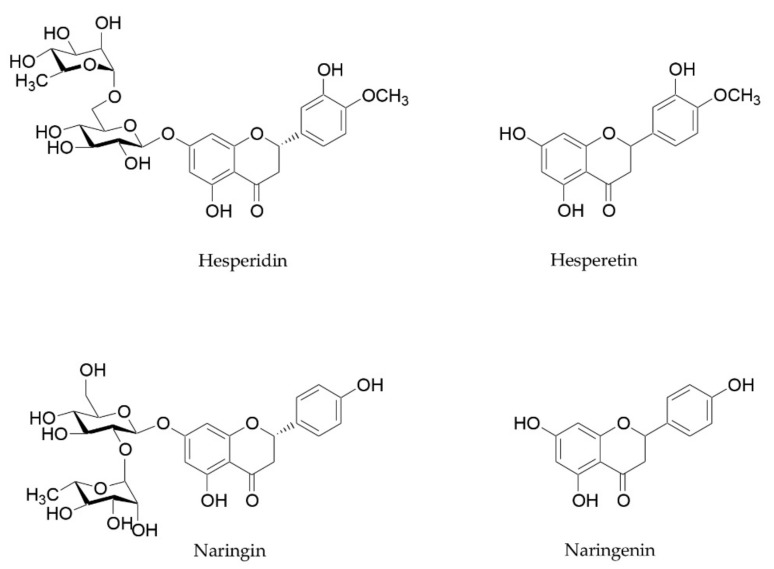
Chemical structure of hesperidin, naringin, and their aglycones hesperetin and naringenin.

*Citrus* flavanones, especially hesperidin and naringin, and their metabolites are also able to improve microbiome composition, reducing GI inflammation levels and strengthening intestinal barrier function [56,93]. These beneficial properties have already been reviewed by Stevens and coworkers [93]. Hesperidin and naringin are two flavanones glycosides, whose aglycones hesperetin and naringenin are attached to disaccharides consisting of glucose and rhamnose (Figure 3). These two flavanones reach the small intestine and the colon unchanged, and they are metabolized into their aglycones by microbiota [94]. These metabolites, in turn, are able to influence the microbiota composition and exert beneficial effects on intestinal barrier function and anti-inflammatory activity. Different studies focused on their ability to inhibit the growth of pathogens, to increase beneficial bacteria such as *Bifidobacterium* and *Lactobacillus* species, and to stimulate the production of SCFAs [93].

Büyüktuncel assessed the naringin and hesperidin content in some orange, grapefruit, and tangerine juices by means of the HPLC method. The highest content of naringin was verified in a natural grapefruit juice (323.07 ± 3.45 mg/L), followed by commercial grapefruit juices and natural tangerine juice, with a content of 56.75 ± 2.01 and 5.39 ± 0.73 mg/L, respectively. Naringin was not detected in commercial and natural orange juice samples and in commercial tangerine juice. For what concerns hesperidin, the highest amount of standard was detected in natural orange juice and in natural tangerine juice (221.70 ± 3.84 and 142.46 ± 2.62 mg/L). Lower amounts of hesperidin were detected in commercial orange juice, commercial tangerine juice, and natural grapefruit juice (26.85 ± 1.82, 12.13 ±1.10, and 5.53 ± 0.87 mg/L) [95]. Similar results were reported by Ribeiro and coworkers, who detected the highest amount of naringin (476.82 µg/mL) in a fresh-pressed grapefruit followed by commercial grapefruit juices and a fresh-pressed orange (209.68 µg/mL) [96]. The phenolic constituents of Citrus fruits juice were also investigated by Belajovà and Suhaj. The naringin and hesperidin content of orange filtered juices and nectars was compared. Data showed that in each analyzed sample, the amount of hesperidin was higher in filtered juices than in nectar, while for what concerns the naringin amount, nectar always showed the highest content of this flavonoid [97].

The impact of a daily orange juice consumption on gut microbiota was investigated by Fidelix and coworkers in a controlled clinical trial: 10 women with a regular diet started consumption of 300 mL/d of orange juice for 60 days and, successively, others 30 days of washout without orange juice. The microbiota composition and the lipid and sugar profiles were monitored. Experimental evidence showed an increase in *Lactobacillus* spp., *Akkermasia* spp., and *Ruminococcus* spp. bacteria, generating a modulatory effect on gut microbiota [65]. Lima and coworkers also tested the effects of orange juice consumption. A controlled clinical trial was performed on 10 healthy women after 2 months of commercial pasteurized orange juice dietary supplementation. The orange juice consumption positively influenced the microbiota, increasing the fecal composition of *Bifidobacterium* spp. and *Lactobacillus* spp. [66].

Moreover, the daily supplementation with two orange juices with different flavanone content for seven days in healthy volunteers resulted in microbiota composition shifts, the most notable one being an increase in the abundance of Clostridia operational taxonomic units from *Mogibacteriaceae*, *Tissierellaceae*, *Veillonellaceae*, *Odoribacteraceae*, and *Ruminococcaceae* families [67].

As fruits belonging to the *Citrus* genus are rich in flavanones, especially in hesperidin [98], the effects of this flavonoid on gut microbiota were recently assessed. The oral administration of hesperidin was able to change the microbiota trim in healthy rats, as demonstrated by Estruel-Amades and colleagues [99]. The treatment induced an increase in bacteria and IgA-coated bacteria, with changes in microbiota composition, mainly the *Lactobacillus* proportion, and it was also able to increase the IgA content in the small intestine. These results demonstrated the immunomodulatory action of hesperidin on the gut-associated lymphoid tissue.

‘Ougan’ mandarin (*Citrus reticulata* cv. suavissima) is another fruit belonging to the *Citrus* genus, which has been recently investigated for its beneficial properties on the intestinal tract. It is a characteristic Chinese mandarin cultivar, local to the Zhejiang province, whose peel has been employed as a traditional Chinese medicine for a long time [100]. Guo and colleagues tested the beneficial effects of lyophilized juice from fresh fruits in a high-fat diet (HFD)-fed C57BL/6J mice [69]. A dose of 20 mL kg^−1^ daily was administered, accounting for about 2.0–2.3 mg of total flavonoids per mouse per day and corresponding to a dose of 5.3–6.1 mg of total flavonoids per kg of human body weight. Ougan mandarin supplementation increased the gut microbiota diversity, causing a reduction in the relative abundance of phylum *Erysipelatoclostridiaceae* phylum and *Erysipelatoclostridium* genus and an increase in SCFA-producing bacteria *Blautia*. The effects of a lactic acid bacteria fermented Ougan juice were evaluated as well.

The anti-inflammatory properties of fruits belonging to this genus have also been investigated. He and coworkers verified the effectiveness of *Citrus aurantium* L. dried fruits and its main flavonoids on trinitrobenzene sulfonic-acid (TNBS)-induced IBD in rats. Dried fruits, orally administered at 125 to 500 mg/kg, decreased colitis inflammatory cell infiltration and inflammatory cytokine levels and ameliorated weight loss and symptoms of diarrhea. The effects of *C. aurantium’s* main bioactive components, naringenin, nobiletin, and hesperetin, were assessed as well. These molecules showed anti-inflammatory effects on lipopolysaccharide-induced RAW 264.7 cells and the two compounds naringenin and nobiletin also showed inhibitory effects on isolated jejunum contraction [68].

Moreover, the controlled clinical trial described by Fidelix and colleagues demonstrated that the orange juice consumption (300 mL/d of orange juice for 60 days in 10 women with a regular diet) induced a downside effect of an increase in glycemia and lipid profiles [65]. A beneficial role was also reported by Lima and coworkers, who demonstrated that the orange juice supplementation increased biochemical parameters such as glucose and insulin sensitivity and low-density lipoprotein (LDL) cholesterol [66].

### 6.2. Cherries (Prunus Genus)

The blue, purple, and red color of many fruits, such as strawberries, blueberries, blackberries, raspberries, and red grapes, is due to the presence of anthocyanins, a group of water-soluble pigments belonging to the phenolic group with known health-promoting properties. Epidemiologic investigations revealed an association between anthocyanin intake and reduced incidences of some diseases, such as cardiovascular disease, diabetes mellitus, and cancer [101,102,103].

Cherries have been reported to promote many health benefits and are characterized by a high polyphenols content, whose beneficial effects on IBD, such as the ability to modulate gut microbiota and intestinal immune function and to inhibit intestinal inflammation, have been widely reported [49,52,59,60,61,62,63,64]. In contrast, they have a relatively low fiber content compared to other fruits, with limited prebiotic potential. Nevertheless, the high number of polyphenolic molecules undergoes biotransformation in the large intestine through many processes performed by local microbiota [72].

Cyanidin-glycosylrutinoside, quercetin rutinoside, chlorogenic acid, and neochlorogenic acid were identified in cherries juice by means of LC/MS analysis. Mayta-Apaza and coworkers demonstrated that tart cherries (*Prunus cerasus* L.) concentrate juice consumption induced an in vitro increase in *Bacteroides*, which has been related to the polysaccharides content and an increase in *Bifidobacterium*, probably due to chlorogenic acid. The authors also reported the results of an in vivo study conducted on 10 young, healthy volunteers who consumed 8 oz. of juice daily for 5 days. In high-*Bacteroides* individuals, tart cherries juice induced a reduction in *Bacteroides* and *Bifidobacterium* and an increase in *Ruminococcus*, *Lachnospiraceae,* and *Collinsella*. On the other hand, an increase in *Bacteroides* or *Prevotella* and *Bifidobacterium* and a decrease in *Lachnospiraceae*, *Ruminococcus,* and *Collinsella* was observed in low-*Bacteroides* individuals [70].

Othaim and colleagues investigated the beneficial effects of tart and sweet cherries juices consumption in mice whose diet was supplemented with an increased concentration of juice added to their drinking water for 23 days. Authors showed that the intake of cherries was associated with a positive modulation of the murine gut microbiome: the large consumption was linked to an increase in *Barnesiella* and *Akkermansia*, but it was inversely correlated with the amount of *Bacteroides* [71].

In contrast, Lear and coworkers reported that supplementation with Montmorency cherries in 28 adults did not cause any change in species richness and diversity, and no significant correlation was evidenced with changes in *Bacteroides* and *Faecalibacterium* abundance [72].

### 6.3. Banana (Musa L. Genus)

Fruits contain several different bioactive compounds: phenolics, carotenoids, phytosterols, and vitamins. Banana pulp and peel contain many phenolics such as gallic acid, catechin, epicatechin, tannins, and anthocyanins [104]. This fruit is considered a rich source of indigestible carbohydrates that can exert a potential prebiotic effect. The potential health benefits related to its consumption have been explored. In a randomized controlled clinical trial on 34 healthy women, only the group treated with a pre-meal snack medium banana showed an increase in *Bifidobacterium* levels after 30 and 60 days of intervention. However, this effect was not statistically significant [73].

Green (unripe) bananas also represent a suitable source of amylase-resistant starch (ARS), a plant prebiotic considered as a type of dietary fiber. ARS is not digested in the small intestine of humans, but it is able to reach the colon, where it is fermented by resident bacteria into SCFAs, butyrate, propionate, and acetate. SCFAs stimulate salt and water absorption and exert a trophic effect [74,105,106]. Moongngarm assessed the content of resistant starch in green banana fruits (*Musa sapientum* L.): it ranged between 35.14% and 45.87% [105].

Rabbani and coworkers reported a cluster randomized field trial conducted on about 3000 children in Bangladesh. The authors demonstrated that children with gut inflammation and diarrhea receiving green bananas for 14 days had significantly improved rates by day 10 [74].

Similar results were evidenced in a double-blind trial involving 62 children receiving green bananas and pectin. Results demonstrated that both green banana and pectin were effective in the dietary management of persistent diarrhea in children, as they significantly reduced vomiting, stool, and diarrhea duration and the need for oral rehydration solution [107].

Moreover, Cassettari and colleagues described a randomized study involving 80 children and adolescents and assessed the effects of the combination of green banana consumption and laxatives for the treatment of chronic constipation. It was observed that banana biomass represented a functional food and an adjunct therapy against constipation in children and adolescents [75].

### 6.4. Apple (Malus Mill. Genus)

Apple is one of the most consumed fruits all over the world. It is rich in vitamin C, vitamin A, fibers and represents a suitable source of polyphenols. Apple fruits are a rich source of flavonols such as epicatechin, quercetin, and rutin [98]. Many of these antioxidant compounds reach the large intestine, where they are fermented in bioactive compounds with many health benefits and that positively influence microbiome diversity and composition [108,109]. Prebiotic dietary fiber, pectin, and apple polyphenols may reduce metabolic endotoxemia through the potential modification of the intestinal microbiota by improving gut barrier function and reducing intestinal permeability. Microbiota-derived polyphenol metabolites may also be responsible for these anti-inflammatory effects. In vitro and in vivo experiments indicated that polyphenols found in apples may reduce intestinal inflammation in humans after microbial degradation suggesting beneficial effects, locally, at the intestinal level [110]. Dihydroxylated phenolic acids, derived from the microbial degradation of phenolic acids, mainly 3,4-dihydroxyphenylpropionic acid and 3,4-dihydroxyphenylacetic acid, significantly inhibited the secretion of proinflammatory cytokines including, TNF-α, IL-1β, and IL-6 in LPS stimulated peripheral blood mononuclear cells from 6 healthy volunteers [111].

There are only a few human studies focusing on the effects of apples on gut microbiota. In a small-scale intervention study (n = 8), 2 apples per day for 2 weeks significantly increased fecal *Bifidobacteria* while reducing *Enterobacteriaceae* and lecithinase-positive *Clostridia*, including *C. perfringens* [76].

Ravn-Haren and colleagues described a recent study involving 23 healthy volunteers whose diet was supplemented with whole apple (variety ‘Shampion’), apple pomace, and juice consumption for 4 weeks. The treatment lowered fecal pH and resulted in differences in denaturing gradient gel electrophoresis (DGGE) profile. However, a potential modulation of the gut microbiota population was not confirmed by quantitative PCR [77].

### 6.5. Blueberry and Cranberry (Vaccinium Genus)

Red fruits such as blueberries, mulberries, blackberries, or red grapes are fruits particularly high in anthocyanin content. As reported by Fang, red grapes (*Vitis* spp.) present a high content of anthocyanins such as delphinidin-3-glucoside, cyanidin-3-glucoside, malvidin-3-glucoside, peonidin-3-glucoside, malvidin-3-coumaryl-glucoside, malvidin-3-acetyl-glucoside, petunidin-3-glucoside [102]. According to Williamson, a portion of blackberries (100 g) contains about 170 mg of anthocyanins [98].

There is growing evidence from animal studies and human clinical trials that diets rich in anthocyanins protect against inflammation and increased gut permeability, as well as improve colon health through their ability to alter bacterial metabolism and the microbial milieu within the intestines.

In the lumen of the large intestine, unabsorbed anthocyanins are exposed to microbiota-mediated biotransformation, which includes three significant conditions: hydrolysis (breaking glycosidic linkages), fission (cleaving heterocycle), and demethylation. Bacterial species that carry corresponding β-glucosidase, β-glucuronidase, α-rhamnosidase, or demethylase such as *Clostridium* spp., *Butyrivibrio* spp., *Lactobacillus* spp., *B. fragilis*, and *B. ovatus*, etc., are actively involved in this process [112].

Many studies evidenced that dietary anthocyanins and their metabolites exert potential health benefits via modulation of the gut microbiome and by increasing SCFAs levels [113].

Blueberries show a high content of fermentable fibers and anthocyanins [114]. In 2011, Vendrame and colleagues evaluated the effects of the consumption of wild blueberry (*Vaccinium angustifolium* Ait.) drink on human gut microbiota. Twenty healthy male volunteers were randomly divided into two groups. The first group received every day a wild blueberry drink (25 g of wild blueberry powder in 250 mL of water) for 6 weeks, in addition to the habitual diet. After a six-week washout period, they received a daily placebo drink for further 6 weeks. The opposite sequence was followed by individuals in the second group. It was observed that *Bifidobacterium* spp. significantly increased following blueberry treatment, suggesting that the regular consumption of a wild blueberry drink can positively affect intestinal microbiota [78].

Interestingly, Paturi and coworkers evaluated the capacity of dietary blueberry (*Vaccinium corymbosum* L. hybrid “Marimba,” “Misty,” and “O’Neal”) and broccoli supplementation to modify microbiota composition in an IBD mouse model in order to reduce colon inflammation. Mice were randomly divided into three experimental groups, control, mice fed with blueberry (200 g/kg), and mice fed with broccoli for 21 weeks. Both blueberries and broccoli supplementation altered the composition and the metabolism of the cecal microbiota and colon morphology [81].

The beneficial properties of a lowbush wild blueberries-enriched diet were further investigated by Lacombe and colleagues, who verified their effects on gut microbiota in Sprague-Dawley rats. The murine model is a powerful tool to study population dynamics, even if the microbiome differs from humans. In this study, a significant increase in the relative abundance of the *Bifidobacteriaceae* and *Coriobacteriaceae* family compared to the control diet was observed [79].

More recently, Lee and coworkers confirmed these health beneficial properties. The effects of dietary supplementation with blueberry were examined on male Wistar rats, and compositional changes in the gut microbiota were observed, with an increase in *Gammaproteobacteria* abundance [80].

Another interesting study focuses on the beneficial effects of *Vaccinium bracteatum* Thunb, commonly known as the oriental blueberry. This evergreen shrubby tree grows in the East and the South of China, Korea, Japan, Malaysia, and Indonesia. The fruits of this plant have been considered a healthy food for centuries [115]. Song and coworkers assessed the effects on the gut microbiota of an extract prepared from *V. bracteatum* fruits. The extract obtained with a hydroalcoholic solution was concentrated, eluted in a XAD-7HP column, and then lyophilized. C57BL/6J mice fed with a high-fat diet were used in this study. For 14 weeks, animals received daily 150 mg/kg body weight of *V. bracteatum* sample in a saline vehicle by intragastric administration, a dose that has been calculated equivalent to 0.72 g/day for 60 kg person. Oriental blueberry supplementation was able to reduce the high-fat diet-induced body weight gain and insulin resistance index. Effects on gut microbiota composition were assessed as well: *V. bracteatum* was able to increase the abundances of *Verrucomicrobia* and *Bacteroidetes* and decrease that of *Proteobacteria* and *Firmicutes* compared with the HFD group [82].

The beneficial effects of cranberry (*Vaccinium macrocarpon* Aiton) were investigated as well. Cai and colleagues evaluated the beneficial effects of this fruit on a dextran sulfate sodium (DSS)-induced colitis mouse model. Diet was supplemented with 1.5 w/w freeze-dried whole cranberry powder, which was calculated to be equivalent to about an oral dose of 65 g of fresh fruit per day for human dietary consumption in a 60 kg adult. A significant decrease in the severity of colitis and the colonic levels of proinflammatory cytokines compared to the positive control group was observed. The anti-inflammatory effects exerted by cranberry supplementation were related to its ability to alleviate DSS-induced changes in the gut microbiota composition: cranberry treatment partially reversed the change of gut microbiota in colitic mice by an increase in the abundance of potentially beneficial bacteria, such as *Lactobacillus* and *Bifidobacterium*, and a decrease in potentially harmful bacteria, such as *Sutterella* and *Bilophila* [83].

### 6.6. Mulberry (Morus Genus)

Black mulberries (*Morus nigra* L.) are a rich source of polyphenols, flavonoids, and anthocyanins that confer antioxidant and anti-inflammatory activities [116].

Zhang and colleagues identified some non-anthocyanin phenolics, procatechuic acid, chlorogenic acid, taxifolin, rutin, and quercetin, in two mulberry cultivars (*Morus alba* L.). For all these molecules, some beneficial effects on IBD have been reported: the first three compounds and quercetin were demonstrated to ameliorate experimental colitis [117,118,119,120], while rutin showed gastroprotective effects on induced ulcers in rats [121]. In particular, rutin was the most abundant compound identified through HPLC-DAD, with values of 111.38 and 90.79 µg/g for da-10 and hongguo cultivars, respectively [122]. According to Kim and Chung, cyanidin-3-rutinoside, identified by LC-MS, was the most abundant compound in *Morus alba* juice [123].

Qian and colleagues investigated the efficacy of mulberry fruit extracts supplementation in mice with DSS-induced acute colitis. A significant improvement was observed in mice fed with mulberry extract. In mice fed with a 3% DSS diet for 9 days, a 10% loss in their initial body weight, 35% shortening in colon length, and 40% increase in spleen weight were detected. All these negative effects were significantly ameliorated by feeding mice with 5% or 10% mulberry fruit extracts, which also significantly prevented DSS-induced severe injuries in colon crypts [84].

Wang and Hatabu investigated the beneficial role of a mulberry juice freeze-dried powder supplementation on DSS-induced acute colitis in male BALB/c mice. Animals were fed with a supplemented diet for 28 days. Results showed that mulberries ameliorated colitis induced by DSS through a gut microbial flora change [85].

Moreover, Wang and coworkers also studied three white mulberry leaf polysaccharides (SY01-21, SY01-22, SY01-23): results showed that SY01-23 was able to modulate gut microbiota by improving *Bacteroides* colonization [124].

### 6.7. Strawberry (Fragaria) and Raspberry (Rubus)

Kanodia and coworkers tested the potential health benefits of the ethanolic extract of wild strawberry (*Fragaria vesca* L.) fruits on acetic acid-induced IBD in rats. Animals were treated with 500 mg/kg of sample for five days. Obtained results demonstrated that strawberry extract was able to improve colon architecture and reduce tissue oxidative stress, with a significant improvement of superoxide dismutase (SOD) and catalase (CAT) tissue levels. The significant amelioration of experimentally induced IBD was in part related to the antioxidant and anti-inflammatory properties of *F. vesca* extract [86].

Montrose and colleagues reported the anti-inflammatory activity of freeze-dried black raspberry powder on DSS-induced ulcerative colitis in C57BL/6J mice. Animals were fed with a diet containing 5% or 10% raspberry sample for 7–14 days, after which the extent of the colonic injury was assessed. A strong anti-inflammatory activity, with a reduction in colonic shortening and ulceration and the suppression of the tissue levels of different proinflammatory cytokines, including TNF-α and interleukin 1β, were observed [87].

Moreover, Chen and colleagues reviewed a number of preclinical and clinical pilot studies concerning the preventive activity of strawberry, black raspberry, and their polyphenol components on colorectal cancer chemoprevention in IBD [125].

### 6.8. Goji (Lycium Genus)

Ding and coworkers investigated the effects of an extract from goji (*Lycium barbarum* L.) on immunoregulation and gut microbiota dysbiosis in cyclophosphamide (CTX)-induced mice [88]. The dried fruits were pulverized, and polysaccharides were properly extracted and tested on CTX-induced mice. Treated mice were administered with 50, 100, and 200 mg kg^−1^ d^−1^ of the sample by intragastric gavage for 9 days. It was observed that *L. barbarum* polysaccharides administration was able to enhance the production of immune-related cytokines and prevent hepatotoxicity in CTX-induced mice. Moreover, goji polysaccharides were demonstrated to promote the production of SCFAs and modulate gut microbiota composition, as an increase in the abundance of *Bacteroidaceae*, *Lactobacillaceae*, *Prevotellaceae,* and *Verrucomicrobiaceae* was observed.

Interestingly, Huang and coworkers investigated the beneficial effects of a natural ascorbic acid derivative from the fruits of *L. barbarum*: the 2-*O*-*β*-D-glucopyranosyl-L-ascorbic acid (AA-2βG). This compound demonstrated beneficial effects on DSS-induced colitis in mice, inducing an improvement of serum physiological and biochemical indicators and a reduction in colitis disease activity index and inflammatory cytokines. Moreover, it was able to promote the production of SCFAs and modulate the gut microbiota composition [126]. A subsequent study by the same authors showed that AA-2βG also exerted an immunomodulatory effect on CTX-treated BALB/c mice [127].

An interesting study dealt with the beneficial properties of anthocyanins from the fruits of black goji (*Lycium ruthenicum* Murray). The fruits were extracted with a hydroalcoholic solution, and the obtained extract was purified with an AB-8 macroporous resin column in order to afford the anthocyanins. The beneficial properties of this anthocyanin extract from black goji fruit were verified on DSS-induced colitis in C57BL/6 mice. Animals received a dose of 200 mg/kg/d for 8 days. *L. ruthenicum* was able to decrease the expression of proinflammatory cytokines, such as TNF-α, IL-6, IL1β, and PGE2. The supplementation also influenced gut microbiota, as the DSS-induced decrease in the relative abundances of *Porphyromonadaceae*, *Rikenellaceae,* and *Prevotellaceae* was reversed [89].

### 6.9. Pomegranate (Punica Genus)

Pomegranate (*Punica granatum* L.) is native to Iran and cultivated throughout the Mediterranean region, Southeast Asia, and in California and Arizona in the United States [128,129]. Song and colleagues evaluated the in vivo microbiota-modulating effects of a polyphenol-rich extract from pomegranate fresh fruits. C57BL/6J mice fed with a high-fat diet received 200 mg/kg daily of pomegranate supplementation by intragastric administration for 14 weeks. The treatment induced some changes in the gut microbiota composition, inducing an increased abundance of *Akkermansia muciniphila*, *Bacteroides acidifaciens*, *Mucispirillum schaedleri,* and other species. Moreover, this supplementation was able to significantly reduce HFD-induced weight gain and insulin resistance compared to HFD mice [90].

### 6.10. Noni Fruit (Moringa Genus)

Noni fruit (*Moringa citrifolia* L.) is an evergreen shrub whose ripe fruit has been used in tropical regions as both food and popular medicine [130,131]. Products derived from noni are commercialized, and they are increasingly distributed all over the world [132]. Yang and coworkers reported the in vivo beneficial properties of a freeze-dried polysaccharide extract obtained from sliced, dehydrated noni fruits. Male Sprague-Dawley rats fed with a high-fat diet received a supplementation equal to 100 mg per kg bw for 5 weeks. The treatment was demonstrated to improve cecal SCFA production and to modulate gut microbiota. Improved levels of *Lactobacillus*, *Ruminococcaceae,* and *Parasutterella*, and decreased levels of *Prevotella_9*, *Collinsella,* and *Bacteroides* were observed [91].

## 7. Conclusions

A number of studies focusing on the potential beneficial effects of vegetable foods, both plant-derived dietary fibers and plant phytochemicals, on gut microbiota [48,49,133] demonstrated that phenolic compounds and other constituents are able to improve gut health and could be useful in the treatment of gastrointestinal disorders. A relationship has been reported between gut microbiota and inflammatory diseases, among which IBD. The role of different factors, such as the increase in *Proteobacteria* and the decrease in the *Firmicutes* phyla, has been demonstrated. The anti-inflammatory effects exerted by SCFAs and a decrease in these metabolites in IBD patients have been observed as well. The beneficial properties of dietary phytochemicals are linked to the ability of these compounds to affect a number of physiological processes such as lipid and glucose metabolism and immune homeostasis.

In this review, both in vitro and in vivo studies concerning the potential beneficial effects of fruits or their juices consumption have been reported. Citrus fruits and blueberries, particularly, appear to be the most investigated fruits as regards their potential beneficial properties on gut microbiota. Many investigated fruit plant species have been demonstrated to enhance the growth of beneficial bacteria strains and to reduce the presence of pathogenic bacteria.

Some negative results have also been described. The dietary supplementation of apples, apple pomace, and juice described by Ravn-Haren and colleagues, for example, did not show a modulation on gut microbiota population in healthy volunteers [77].

Moreover, for some of the studies here reported, animal models such as mice or rats were used to study how fruit samples could influence gut microbiota. However, rodents differ from humans both in terms of gut microbiota composition and structure of the intestine [134]. As a consequence, some of the relevant findings here reported remain to be confirmed.

## Figures and Tables

**Table 1:** Fruit species investigated for their potential beneficial role on gut microbiota.

Plant Genus	Plant Species	Sample/Extract	Study	Animal Model/Participants	Treatment	Results	Ref
Citrus	Orange (*Citrus* *sinensis* L.)	Juice (‘Pera Rio’ variety)	In vivo	10 women	Consumption of 300 mL/d for 60 days	Increased levels of *Lactobacillus*, *Akkermasia*, and *Ruminococcus* spp.	[65]
		Juice	In vivo	10 healthy women	2 months of commercial pasteurized orange juice consumption	Increased fecal composition of *Bifidobacterium* and *Lactobacillus* spp.	[66]
		Juice (‘Cara Cara’ and ‘Bahia’)	In vivo	21 healthy volunteers (18–45 years)	Daily supplementation of two orange juices with different flavanone content for 7 days	Increased abundance of *Clostridia* operational taxonomic units from *Mogibacteriaceae*, *Tissierellaceae*, *Veillonellaceae, Odoribacteraceae,* and *Ruminococcaceae* families	[67]
	Bitter orange (*C. aurantium* L.)	Dried fruits	In vivo	Trinitrobenzene sulfonic-acid (TNBS)-induced IBD in rats	From 125 to 500 mg/kg in rats with IBD	Decreased colitis inflammatory cell infiltration and inflammatory cytokine levels and ameliorated weight loss and diarrhea symptoms	[68]
	‘Ougan’ mandarin (*C. reticulata* cv. suavissima)	Lyophilized juice	In vivo	Fifty high-fat diet (HFD)-fed C57Bl/6J male mice	20 mL kg^−1^ daily	Reduced abundance of phylum *Erysipelatoclostridiaceae* and remarkably increase in SCFA-producing bacteria *Blautia*	[69]
*Prunus*	Tart cherry (*Prunus cerasus* L.)	Juice	In vitro	-	-	Increase in *Bacteroides* and *Bifidobacterium*	[70]
		Juice	In vivo	10 young, healthy participants (5 males, 5 females)	8 oz. of juice daily for 5 days	Increase in *Ruminococcus*, *Lachnospiraceae*, and *Collinsella* in high-*Bacteroides* individuals. Increase in *Bacteroides* in low-*Bacteroides*	[70]
		Juice	In vivo	45 mice	juice added to drinking water for 23 days	Increase in *Barnesiella* and *Akkermansia*	[71]
		Concentrate	In vivo	28 participants (40–60 years)	60 mL per day for 4 weeks	Supplementation did not alter gut microbiome	[72]
*Prunus*	Sweet cherry (*Prunus avium* L.)	Juice	In vivo	45 mice	Increased concentration of juice added to drinking water for 23 days	Increase in *Barnesiella* and *Akkermansia*	[71]
*Musa*	Banana	Fruit and flavored drink	In vivo	34 healthy women (19–45 years)	Pre-meal snack (fruit or banana-flavored drink) twice a day	Not statistically significant increase in *Bifidobacterium* levels after 30 and 60 days of intervention	[73]
		Cooked green banana mixed with rice flour	In vivo	62 children	250 g/L of cooked green banana for 7 days	Reduced vomiting, stool, and diarrheal duration and reduced need for oral rehydration solution	[74]
		Cooked fruits	In vivo	80 children and adolescents	-	Green banana biomass resulted advantageous as an adjunct therapy on functional constipation, mainly for reducing doses of laxatives	[75]
*Malus*	Apple	Fresh fruit	In vivo	8 healthy male volunteers (21–60 years)	2 apples per day for 2 weeks	Increased fecal *Bifidobacteria*; reduced *Enterobacteriaceae* and lecithinase-positive *Clostridia*, including *C. perfringens*	[76]
		Apples, apple pomace, and juice	In vivo	23 healthy volunteers	5 × 4 weeks dietary crossover study: whole apples (550 g/day), apple pomace (22 g/day), apple juices (500 mL/day)	A modulation of the gut microbiota population was not confirmed	[77]
*Vaccinium*	Blueberry (*V. angustifolium* Ait.)	Wild blueberry drink	In vivo	20 healthy male volunteers	Wild blueberry drink (25 g of wild blueberry powder in 250 mL of water) for 6 weeks and daily placebo drink for further 6 weeks	Increased *Bifidobacterium* spp.	[78]
		Blueberry powder	In vivo	Sprague-Dawley rats	Control diet + 8% *w/w *blueberry powdersubstituting for dextrose	Increased relative abundance of *Bifidobacteriaceae* and *Coriobacteriaceae* family	[79]
		Blueberry powder	In vivo	24 male Wistar rats	10 g freeze-dried blueberry powder/100 g diets for 8 weeks	Increased *Gammaproteobacteria* abundance	[80]
	Blueberry (*V. corymbosum* L.)	Fruit (hybrid “Marimba,” “Misty”, “O’Neal”)	In vivo	IBD mouse model	200 g/kg for 21 weeks	Altered composition and metabolism of the cecal microbiota and colon morphology	[81]
	Oriental blueberry (*V. bracteatum* Thunb)	Polyphenol-rich fruit extract	In vivo	High-fat diet (HFD)-induced obese mice	150 mg/kg body weight for 14 weeks	Reduced HFD induced body weight gain and insulin resistance index; increased abundances of *Verrucomicrobia* and *Bacteroidetes*, and decreased *Proteobacteria* and *Firmicutes*	[82]
	Cranberry (*V. macrocarpon* Aiton)	Freeze-dried whole cranberry powder	In vivo	Dextran sulfate sodium (DSS)-induced acute colitis in mice	Diet + 1.5% (*w/w*) freeze-dried whole cranberry powder	Decrease in the severity of colitis and of proinflammatory cytokines levels. Increase in the abundance *Lactobacillus* and *Bifidobacterium* and decrease in *Sutterella* and *Bilophila*	[83]
*Morus*	Mulberry (*Morus alba* L.)	Mulberry fruits extracts	In vivo	DSS-induced acute colitis in mice	5% or 10% mulberry extracts for 3 months	Amelioration of colitis and prevention of severe injuries in colon crypts	[84]
		Mulberry juice freeze-dried powder	In vivo	DSS-induced acute colitis in BALB/c mice	28 days	Mitigation of DSS-induced acute colitis by changing the gut microbial flora and by improving mucosal conditions	[85]
*Fragaria*	Strawberry (*Fragaria vesca* L.)	Ethanolic extract	In vivo	Acetic acid-induced IBD in rats	500 mg/kg for 5 days	Improved colon architecture and reduced tissue oxidative stress, with a significant improvement of superoxide dismutase and catalase tissue levels	[86]
*Rubus*	Raspberry	Freeze-dried black raspberry powder	In vivo	DSS-induced ulcerative colitis in C57BL/6J mice	5% or 10% raspberry sample for 7–14 days	Anti-inflammatory activity, with reduction in colonic shortening and ulceration and suppression of different proinflammatory cytokines	[87]
*Lycium*	Goji (*L. barbarum* L.)	Extract from dried fruits	In vivo	Cyclophosphamide (CTX)-induced mice	50, 100, and 200 mg/kg d^−1^ by intragastric gavage for 9 days	Promoted production of short-chain fatty acids. Increase in *Bacteroidaceae, Lactobacillaceae,* *Prevotellaceae* and *Verrucomicrobiaceae*	[88]
	Black goji (*L. ruthenicum* Murray)	Anthocyanins extract from fruits	In vivo	DSS-induced colitis in C57BL/6 mice	200 mg/kg/d for 8 days	Decrease in the expression of proinflammatory cytokines; reversal of DSS-induced decreases in relative abundances of *Porphyromonadaceae*, *Rikenellaceae* and *Prevotellaceae*	[89]
*Punica*	Pomegranate (*P. granatum* L.)	Polyphenol extract from fresh fruits	In vivo	C57BL/6J mice	200 mg/kg daily by intragastric administration for 14 weeks	Changes in gut microbiota composition. Increased abundance of *Akkermansia muciniphila, Bacteroides acidifaciens, Mucispirillum schaedleri,* and other species	[90]
*Moringa*	Noni (*M. citrifolia* L.)	Freeze-dried polysaccharide extract from dehydrated fruits	In vivo	Sprague-Dawley rats	100 mg per kg bw for 5 weeks	Improved cecal short-chain fatty acid (SCFA) production; improvement in the levels of *Lactobacillus, Ruminococcaceae*, and *Parasutterella*, and decrement in the levels of *Prevotella_9, Collinsella*, and *Bacteroides*	[91]

## Data Availability

Data sharing is not applicable to this article.

## Abbreviations

The following abbreviations are used in the manuscript:

AA-2βG	2-O-β-D-glucopyranosyl-L-ascorbic acid
CD	Chron’s disease
CTX	cyclophosphamide
DGGE	denaturing gradient gel electrophoresis
DSS	dextran sulfate sodium
ENT	enteral nutritional therapy
EO-IBD	early-onset inflammatory bowel disease
FMT	fecal microbiota transplantation
GI	gastrointestinal
IBD	inflammatory bowel disease
JAK/STAT	Janus kinase/signal transducer and activator of transcription
LDL	low-density lipoproteins
miRNAs	MicroRNAs
n-3 PUFA	n-3 polyunsaturated fatty acids
n-9 PUFA	n-9 polyunsaturated fatty acids
SCFAs	short-chain fatty acids
GPR43	G-protein-coupled receptor 43
SOD	superoxide dismutase
TNBS	trinitrobenzene sulfonic-acid
TNF-α	tumor necrosis factor-alpha
UC	ulcerative colitis
VEO-IBD	very-early-onset inflammatory bowel disease

## References

[1] Kaplan G.G. (2015). The global burden of IBD: From 2015 to 2025. Nat. Rev. Gastroenterol. Hepatol..

[2] Loddo I., Romano C. (2015). Inflammatory bowel disease: Genetics, epigenetics, and pathogenesis. Front. Immunol..

[3] Weimers P., Munkholm P. (2018). The natural history of IBD: Lessons learned. Curr. Treat. Options. Gastro..

[4] Vatn M.H. (2009). Natural history and complications of IBD. Curr. Gastroenterol. Rep..

[5] Mulder D.J., Noble A.J., Justinich C.J., Duffin J.M. (2014). A Tale of two diseases: The history of inflammatory bowel disease. J. Crohns Colitis.

[6] Farzaei M.H., Bahramsoltani R., Abdolghaffari A.H., Sodagari H.R., Esfahani S.A., Rezaei N. (2016). A Mechanistic review on plant-derived natural compounds as dietary supplements for prevention of inflammatory bowel disease. Expert. Rev. Gastroenterol. Hepatol..

[7] Larussa T., Imeneo M., Luzza F. (2017). Potential role of nutraceutical compounds in Inflammatory Bowel Disease. World J. Gastroenterol..

[8] Neurath M.F. (2017). Current and emerging therapeutic targets for IBD. Nat. Rev. Gastroenterol. Hepatol..

[9] Lucendo A.J., De Rezende L.C. (2009). Importance of nutrition in inflammatory bowel disease. World J. Gastroenterol..

[10] Raman M., Ghosh S. (2019). Diet and nutrition in IBD—Progress and gaps. Nutrients.

[11] Wu G.D., Bushmanc F.D., Lewis J.D. (2013). Diet, the human gut microbiota, and IBD. Anaerobe.

[12] Tomasello G., Mazzola M., Leone A., Sinagra E., Zummo G., Farina F., Damiani P., Cappello F., Gerges Geagea A., Jurjus A. (2016). Nutrition, oxidative stress and intestinal dysbiosis: Influence of diet on gut microbiota in inflammatory bowel diseases. Biomed. Pap. Med. Fac. Univ. Palacky Olomouc Czech Repub..

[13] Kaplan G.G., Windsor J.W. (2021). The Four epidemiological stages in the global evolution of inflammatory bowel disease. Nat. Rev. Gastroenterol. Hepatol..

[14] Ananthakrishnan A.N., Bernstein C.N., Iliopoulos D., Macpherson A., Neurath M.F., Ali R.A.R., Vavricka S.R., Fiocchi C. (2018). Environmental triggers in IBD: A review of progress and evidence. Nat. Rev. Gastroenterol. Hepatol..

[15] Mentella M.C., Scaldaferri F., Pizzoferrato M., Gasbarrini A., Miggiano G.A.D. (2020). Nutrition, IBD and gut microbiota: A review. Nutrients.

[16] Guan Q. (2019). A Comprehensive review and update on the pathogenesis of inflammatory bowel disease. J. Immunol. Res..

[17] Khan I., Ullah N., Zha L., Bai Y., Khan A., Zhao T., Che T., Zhang C. (2019). Alteration of gut microbiota in inflammatory bowel disease (IBD): Cause or consequence? IBD treatment targeting the gut microbiome. Pathogens.

[18] Chudy-Onwugaje K.O., Christian K.E., Farraye F.A., Cross R.K. (2019). A state-of-the-art review of new and emerging therapies for the treatment of IBD. Inflamm. Bowel Dis..

[19] Park S.C., Jeen Y.T. (2018). Anti-integrin therapy for inflammatory bowel disease. World J. Gastroenterol..

[20] Salas A., Hernandez-Rocha C., Duijvestein M., Faubion W., McGovern D., Vermeire S., Vetrano S., Casteele N.V. (2020). JAK–STAT pathway targeting for the treatment of inflammatory bowel disease. Nat. Rev. Gastroenterol. Hepatol..

[21] Soendergaard C., Bergenheim F.H., Bjerrum J.T., Nielsen O.H. (2018). Targeting JAK-STAT signal transduction in IBD. Pharmacol. Ther..

[22] Peyrin-Biroulet L., Christopher R., Behan D., Lassen C. (2017). Modulation of sphin-gosine-1-phosphate in inflammatory bowel disease. Autoimmun. Rev..

[23] Thursby E., Juge N. (2017). Introduction to the human gut microbiota. Biochem. J..

[24] Eckburg P.B., Bik E.M., Bernstein C.N., Purdom E., Dethlefsen L., Sargent M., Gill S.R., Nelson K.E., Relman D.A. (2005). Diversity of the human intestinal microbial flora. Science.

[25] Proctor L.M. (2011). The human microbiome project in 2011 and beyond. Cell Host Microbe.

[26] Donaldson G.P., Lee S.M., Mazmanian S.K. (2016). Gut biogeography of the bacterial microbiota. Nat. Rev. Microbiol..

[27] Sun J., Chang E.B. (2014). Exploring gut microbes in human health and disease: Pushing the envelope. Genes Dis..

[28] Umbrello G., Esposito S. (2016). Microbiota and neurologic diseases: Potential effects of probiotics. J. Transl. Med..

[29] Round J.L., Mazmanian S.K. (2009). The gut microbiota shapes intestinal immune responses during health and disease. Nat. Rev. Immunol..

[30] Cryan J.F., Dinan T.G. (2012). Mind-altering microorganisms: The impact of the gut microbiota on brain and behaviour. Nat. Rev. Neurosci..

[31] Weiss G.A., Hennet T. (2017). Mechanisms and consequences of intestinal dysbiosis. Cell. Mol. Life Sci..

[32] Levy M., Kolodziejczyk A.A., Thaiss C.A., Elinav E. (2017). Dysbiosis and the immune system. Nat. Rev. Immunol..

[33] Ajslev T.A., Andersen C.S., Gamborg M., Sørensen T.I.A., Jess T. (2011). Childhood overweight after establishment of the gut microbiota: The role of delivery mode, pre-pregnancy weight and early administration of antibiotics. Int. J. Obes..

[34] Jostins L., Ripke S., Weersma R.K., Duerr R.H., McGovern D.P., Hui K.Y., Lee J.C., Schumm L.P., Sharma Y., Anderson C.A. (2012). Host-microbe interactions have shaped the genetic architecture of inflammatory bowel disease. Nature.

[35] Nishida A., Inoue R., Inatomi O., Bamba S., Naito Y., Andoh A. (2018). Gut microbiota in the pathogenesis of inflammatory bowel disease. Clin. J. Gastroenterol..

[36] Forbes J.D., Chen C.Y., Knox N.C., Marrie R.A., El-Gabalawy H., de Kievit T., Alfa M., Bernstein C.N., Van Domselaar G. (2018). A comparative study of the gut microbiota in immune-mediated in-flammatory diseases—Does a common dysbiosis exist?. Microbiome.

[37] Zhao L. (2013). The gut microbiota and obesity: From correlation to causality. Nat. Rev. Microbiol..

[38] Li W.Z., Stirling K., Yang J.J., Zhang L. (2020). Gut microbiota and diabetes: From correlation to causality and mechanism. World J. Diabetes.

[39] Wang J., Chen W.D., Wang Y.D. (2020). The relationship between gut microbiota and inflammatory diseases: The role of macrophages. Front. Microbiol..

[40] Vinolo M.A., Rodrigues H.G., Nachbar R.T., Curi R. (2011). Regulation of inflammation by short chain fatty acids. Nutrients.

[41] Lavelle A., Sokol H. (2020). Gut microbiota-derived metabolites as key actors in inflammatory bowel disease. Nat. Rev. Gastroenterol. Hepatol..

[42] Ni J., Wu G.D., Albenberg L., Tomov V.T. (2017). Gut microbiota and IBD: Causation or correlation?. Nat. Rev. Gastroenterol. Hepatol..

[43] Barber T.M., Kabisch S., Pfeiffer A.F.H., Weickert M.O. (2020). The health benefits of dietary fibre. Nutrients.

[44] Liu S., Lee I.-M., Ajani U., Cole S.R., Buring J.E., Manson J.E. (2001). Intake of vegetables rich in carotenoids and risk of coronary heart disease in men: The physicians’ health study. Int. J. Epidemiol..

[45] Bazzano L.A., He J., Ogden L.G., Loria C.M., Vupputuri S., Myers L., Whelton P.K. (2002). Fruit and vegetable intake and risk of cardiovascular disease in US adults: The first national health and nutrition examination survey epidemiologic follow-up study. Am. J. Clin. Nutr..

[46] Bazzano L.A., Serdula M.K., Liu S. (2003). Dietary intake of fruits and vegetables and risk of cardiovascular disease. Curr. Atheroscler. Rep..

[47] Liu R.H. (2003). Health benefits of fruit and vegetables are from additive and synergistic combinations of phytochemicals. Am. J. Clin. Nutr..

[48] Cui J., Lian Y., Zhao C., Du H., Han Y., Gao W., Xiao H., Zheng J. (2019). Dietary fibers from fruits and vegetables and their health benefits via modulation of gut microbiota. Compr. Rev. Food Sci. Food Saf..

[49] Klinder A., Shen Q., Heppel S., Lovegrove J.A., Rowland I., Tuohy K.M. (2016). Impact of increasing fruit and vegetables and flavonoid intake on the human gut microbiota. Food Funct..

[50] Gibson G.R. (2004). Fibre and effects on probiotics (the prebiotic concept). Clin. Nutr. Suppl..

[51] Holscher H.D. (2017). Dietary fiber and prebiotics and the gastrointestinal microbiota. Gut Microbes.

[52] Etxeberria U., Fernández-Quintela A., Milagro F.I., Aguirre L., Martínez J.A., Portillo M.P. (2013). Impact of polyphenols and polyphenol-rich dietary sources on gut microbiota composition. J. Agric. Food Chem..

[53] Xu M., Xu X., Li J., Li F. (2019). Association between gut microbiota and autism spectrum disorder: A systematic review and meta-analysis. Front. Psychiatry.

[54] Thumann T.A., Pferschy-Wenzig E.-M., Moissl-Eichinger C., Bauer R. (2019). The role of gut microbiota for the activity of medicinal plants traditionally used in the European Union for gastrointestinal disorders. J. Ethnopharmacol..

[55] Luo J., Lin X., Bordiga M., Brennan C., Xu B. (2021). Manipulating effects of fruits and vegetables on gut microbiota—A critical review. Int. J. Food Sci. Technol..

[56] Musumeci L., Maugeri A., Cirmi S., Lombardo G.E., Russo C., Gangemi S., Calapai G., Navarra M. (2020). Citrus fruits and their flavonoids in inflammatory bowel disease: An overview. Nat. Prod. Res..

[57] Hossen I., Wu H., Luo T., Mehmood A., Jingyi S., Yanping C., Hongqing W., Zhipeng G., Kaiqi Z., Fang Y. (2019). Phytochemicals and inflammatory bowel disease: A review. Crit. Rev. Food Sci. Nutr..

[58] Somani S.J., Modi K.P., Majumdar A.S., Sadarani B.N. (2015). Phytochemicals and their potential usefulness in inflammatory bowel disease. Phytother. Res..

[59] Pei R., Liu X., Bolling B. (2020). Flavonoids and gut health. Curr. Opin. Biotechnol..

[60] Espín J.C., González-Sarrías A., Tomás-Barberán F.A. (2017). The gut microbiota: A Key factor in the therapeutic effects of (poly)phenols. Biochem. Pharmacol..

[61] Ozdal T., Sela D.A., Xiao J., Boyacioglu D., Chen F., Capanoglu E. (2016). The reciprocal interactions between polyphenols and gut microbiota and effects on bioaccessibility. Nutrients.

[62] Cardona F., Andrés-Lacueva C., Tulipani S., Tinahones F.J., Queipo-Ortuño M.I. (2013). Benefits of polyphenols on gut microbiota and implications in human health. J. Nutr. Biochem..

[63] Valdés L., Cuervo A., Salazar N., Ruas-Madiedo P., Gueimonde M., González S. (2015). The relationship between phenolic compounds from diet and microbiota: Impact on human health. Food Funct..

[64] Davinelli S., Scapagnini G. (2021). Interactions between dietary polyphenols and aging gut microbiota: A review. BioFactors.

[65] Fidélix M., Milenkovic D., Sivieri K., Cesar T. (2020). Microbiota modulation and effects on metabolic biomarkers by orange juice: A controlled clinical trial. Food Funct..

[66] Lima A.C.D., Cecatti C., Fidélix M.P., Adorno M.A.T., Sakamoto I.K., Cesar T.B., Sivieri K. (2019). Effect of daily consumption of orange juice on the levels of blood glucose, lipids, and gut microbiota metabolites: Controlled clinical trials. J. Med. Food..

[67] Brasili E., Hassimotto N.M.A., Del Chierico F., Marini F., Quagliariello A., Sciubba F., Miccheli A., Putignani L., Lajolo F. (2019). Daily consumption of orange juice from *Citrus Sinensis* L. Osbeck Cv. Cara Cara and Cv. Bahia differently affects gut microbiota profiling as unveiled by an integrated meta-omics approach. J. Agric. Food Chem..

[68] He W., Li Y., Liu M., Yu H., Chen Q., Chen Y., Ruan J., Ding Z., Zhang Y., Wang T. (2018). *Citrus Aurantium* L. and its flavonoids regulate TNBS-induced inflammatory bowel disease through anti-inflammation and suppressing isolated jejunum contraction. Int. J. Mol. Sci..

[69] Guo X., Cao X., Fang X., Guo A., Li E. (2021). Inhibitory effects of fermented ougan (*Citrus Reticulata* Cv. Suavissima) juice on high-fat diet-induced obesity associated with white adipose tissue browning and gut microbiota modulation in mice. Food Funct..

[70] Mayta-Apaza A.C., Pottgen E., De Bodt J., Papp N., Marasini D., Howard L., Abranko L., Van de Wiele T., Lee S.-O., Carbonero F. (2018). Impact of tart cherries polyphenols on the human gut microbiota and phenolic metabolites in vitro and in vivo. J. Nutr. Biochem..

[71] Othaim A.A., Marasini D., Carbonero F. (2020). Impact of increasing concentration of tart and sweet cherries juices concentrates on healthy mice gut microbiota. Food Front..

[72] Lear R., O’Leary M., O’Brien Andersen L., Holt C.C., Stensvold C.R., van der Giezen M., Bowtell J.L. (2019). Tart cherry concentrate does not alter the gut microbiome, glycaemic control or systemic inflammation in a middle-aged population. Nutrients.

[73] Mitsou E.K., Kougia E., Nomikos T., Yannakoulia M., Mountzouris K.C., Kyriacou A. (2011). Effect of banana consumption on faecal microbiota: A randomised, controlled trial. Anaerobe.

[74] Rabbani G.H., Larson C.P., Islam R., Saha U.R., Kabir A. (2010). Green banana-supplemented diet in the home management of acute and prolonged diarrhoea in children: A community-based trial in rural Bangladesh. Trop. Med. Int. Health.

[75] Cassettari V.M.G., Machado N.C., de Arruda Lourenção P.L.T., Carvalho M.A., Ortolan E.V.P., Cassettari V.M.G. (2019). Combinations of laxatives and green banana biomass on the treatment of functional constipation in children and adolescents: A randomized study. J. Pediatr..

[76] Shinohara K., Ohashi Y., Kawasumi K., Terada A., Fujisawa T. (2010). Effect of apple intake on fecal microbiota and metabolites in humans. Anaerobe.

[77] Ravn-Haren G., Dragsted L.O., Buch-Andersen T., Jensen E.N., Jensen R.I., Németh-Balogh M., Paulovicsová B., Bergström A., Wilcks A., Licht T.R. (2013). Intake of whole apples or clear apple juice has contrasting effects on plasma lipids in healthy volunteers. Eur. J. Nutr..

[78] Vendrame S., Guglielmetti S., Riso P., Arioli S., Klimis-Zacas D., Porrini M. (2011). Six-week consumption of a wild blueberry powder drink increases *Bifidobacteria* in the human gut. J. Agric. Food Chem..

[79] Lacombe A., Li R.W., Klimis-Zacas D., Kristo A.S., Tadepalli S., Krauss E., Young R., Wu V.C.H. (2013). Lowbush wild blueberries have the potential to modify gut microbiota and xenobiotic metabolism in the rat colon. PLoS ONE.

[80] Lee S., Keirsey K.I., Kirkland R., Grunewald Z.I., Fischer J.G., de La Serre C.B. (2018). Blueberry supplementation influences the gut microbiota, inflammation, and insulin resistance in high-fat-diet-fed rats. J. Nutr..

[81] Paturi G., Mandimika T., Butts C.A., Zhu S., Roy N.C., McNabb W.C., Ansell J. (2012). Influence of dietary blueberry and broccoli on cecal microbiota activity and colon morphology in Mdr1a(-/-) mice, a model of inflammatory bowel diseases. Nutrition.

[82] Song H., Shen X., Chu Q., Zheng X. (2021). *Vaccinium Bracteatum* Thunb. fruit extract reduces high-fat diet-induced obesity with modulation of the gut microbiota in obese mice. J. Food Biochem..

[83] Cai X., Han Y., Gu M., Song M., Wu X., Li Z., Li F., Goulette T., Xiao H. (2019). Dietary cranberry suppressed colonic inflammation and alleviated gut microbiota dysbiosis in dextran sodium sulfate-treated mice. Food Funct..

[84] Qian Z., Wu Z., Huang L., Qiu H., Wang L., Li L., Yao L., Kang K., Qu J., Wu Y. (2015). Mulberry fruit prevents LPS-induced NF-ΚB/PERK/MAPK signals in macrophages and suppresses acute colitis and colorectal tumorigenesis in mice. Sci. Rep..

[85] Wang Y., Hatabu T. (2019). Mulberry juice freeze-dried powder attenuates the disease severity by the maintaining of colon mucosa in mice with DSS-induced acute colitis. Biosci. Biotechnol. Biochem..

[86] Kanodia L., Borgohain M., Das S. (2011). Effect of fruit extract of *Fragaria Vesca* L. on experimentally induced inflammatory bowel disease in albino rats. Indian J. Pharmacol..

[87] Montrose D.C., Horelik N.A., Madigan J.P., Stoner G.D., Wang L.-S., Bruno R.S., Park H.J., Giardina C., Rosenberg D.W. (2011). Anti-inflammatory effects of freeze-dried black raspberry powder in ulcerative colitis. Carcinogenesis.

[88] Ding Y., Yan Y., Chen D., Ran L., Mi J., Lu L., Jing B., Li X., Zeng X., Cao Y. (2019). Modulating effects of polysaccharides from the fruits of *Lycium Barbarum* on the immune response and gut microbiota in cyclophosphamide-treated mice. Food Funct..

[89] Peng Y., Yan Y., Wan P., Chen D., Ding Y., Ran L., Mi J., Lu L., Zhang Z., Li X. (2019). Gut microbiota modulation and anti-inflammatory properties of anthocyanins from the fruits of *Lycium Ruthenicum* Murray in dextran sodium sulfate-induced colitis in mice. Free Radic. Biol. Med..

[90] Song H., Shen X., Chu Q., Zheng X. (2021). Pomegranate fruit pulp polyphenols reduce diet-induced obesity with modulation of gut microbiota in mice. J. Sci. Food Agric..

[91] Yang X., Mo W., Zheng C., Li W., Tang J., Wu X. (2020). Alleviating effects of noni fruit polysaccharide on hepatic oxidative stress and inflammation in rats under a high-fat diet and its possible mechanisms. Food Funct..

[92] Zhao C., Wang F., Lian Y., Xiao H., Zheng J. (2020). Biosynthesis of *Citrus* flavonoids and their health effects. Crit. Rev. Food Sci. Nutr..

[93] Stevens Y., Rymenant E.V., Grootaert C., Camp J.V., Possemiers S., Masclee A., Jonkers D. (2019). The intestinal fate of Citrus flavanones and their effects on gastrointestinal health. Nutrients.

[94] Xiao J. (2017). Dietary flavonoid aglycones and their glycosides: Which show better biological significance?. Crit. Rev. Food Sci. Nutr..

[95] Büyüktuncel S. (2017). Fast Determination of naringin and hesperidin in natural and commercial *Citrus* juices by HPLC method. Asian J. Chem..

[96] Ribeiro I.A., Ribeiro M.H.L. (2008). Naringin and naringenin determination and control in grapefruit juice by a validated HPLC method. Food Control..

[97] Belajová E., Suhaj M. (2004). Determination of phenolic constituents in *Citrus* juices: Method of high performance Liquid chromatography. Food Chem..

[98] Williamson G. (2017). The role of polyphenols in modern nutrition. Nutr Bull..

[99] Estruel-Amades S., Massot-Cladera M., Pérez-Cano F.J., Franch À., Castell M., Camps-Bossacoma M. (2019). Hesperidin effects on gut microbiota and gut-associated lymphoid tissue in healthy rats. Nutrients.

[100] Zhang J., Wu Y., Zhao X., Luo F., Li X., Zhu H., Sun C., Chen K. (2014). Chemopreventive effect of flavonoids from ougan (*Citrus Reticulata* Cv. Suavissima) fruit against cancer cell proliferation and migration. J. Funct. Foods.

[101] Li D., Wang P., Luo Y., Zhao M., Chen F. (2017). Health benefits of anthocyanins and molecular mechanisms: Update from recent decade. Crit. Rev. Food Sci. Nutr..

[102] Fang J. (2015). Classification of fruits based on anthocyanin types and relevance to their health effects. Nutrition.

[103] Khoo H.E., Azlan A., Tang S.T., Lim S.M. (2017). Anthocyanidins and anthocyanins: Colored pigments as food, pharmaceutical ingredients, and the potential health benefits. Food Nutr. Res..

[104] Sidhu J.S., Zafar T.A. (2018). Bioactive compounds in banana fruits and their health benefits. Food Qual. Saf..

[105] Moongngarm A. (2013). chemical compositions and resistant starch content in starchy foods. Am. J. Agric. Biol. Sci..

[106] Rabbani G.H., Albert M.J., Rahman H., Chowdhury A.K. (1999). Short-chain fatty acids inhibit fluid and electrolyte loss induced by cholera toxin in proximal colon of rabbit in vivo. Dig. Dis. Sci..

[107] Rabbani G.H., Teka T., Zaman B., Majid N., Khatun M., Fuchs G.J. (2001). Clinical studies in persistent diarrhea: Dietary management with green banana or pectin in Bangladeshi children. Gastroenterology.

[108] Escarpa A., González M.C. (1998). High-performance liquid chromatography with diode-array detection for the determination of phenolic compounds in peel and pulp from different apple varieties. J. Chromatogr. A.

[109] Koutsos A., Tuohy K.M., Lovegrove J.A. (2015). Apples and cardiovascular health—Is the gut microbiota a core consideration?. Nutrients.

[110] Larrosa M., Luceri C., Vivoli E., Pagliuca C., Lodovici M., Moneti G., Dolara P. (2009). Polyphenol metabolites from colonic microbiota exert anti-inflammatory activity on different inflammation models. Mol. Nutr. Food Res..

[111] Monagas M., Khan N., Andrés-Lacueva C., Urpí-Sardá M., Vázquez-Agell M., Lamuela-Raventós R.M., Estruch R. (2009). Dihydroxylated phenolic acids derived from microbial metabolism reduce lipopolysaccharide-stimulated cytokine secretion by human peripheral blood mononuclear cells. Br. J. Nutr..

[112] Selma M.V., Espin J.C., Tomas-Barberan F.A. (2009). Interaction between phenolics and gut microbiota: Role in human health. J. Agric. Food Chem..

[113] Hidalgo M., Oruna-Concha M.J., Kolida S., Walton G.E., Kallithraka S., Spencer J.P., de Pascual-Teresa S. (2012). Metabolism of anthocyanins by human gut microflora and their influence on gut bacterial growth. J. Agric. Food Chem..

[114] Lin Z., Fischer J., Wicker L. (2016). Intermolecular binding of blueberry pectin-rich fractions and anthocyanin. Food Chem..

[115] Fan M., Li T., Li Y., Qian H., Zhang H., Rao Z., Wang L. (2021). *Vaccinium Bracteatum* Thunb. as a promising resource of bioactive compounds with health benefits: An updated review. Food Chem..

[116] De Pádua Lúcio K., Rabelo A.C.S., Araújo C.M., Brandão G.C., de Souza G.H.B., da Silva R.G., de Souza D.M.S., Talvani A., Bezerra F.S., Cruz Calsavara A.J. (2018). Anti-inflammatory and antioxidant properties of black mulberry (*Morus Nigra* L.) in a model of LPS-induced sepsis. Oxid. Med. Cell Longev..

[117] Crespo I., San-Miguel B., Mauriz J.L., Ortiz de Urbina J.J., Almar M., Tuñón M.J., González-Gallego J. (2017). Protective effect of protocatechuic acid on TNBS-induced coli-tis in mice is associated with modulation of the SphK/S1P signaling pathway. Nutrients.

[118] Zhang Z., Wu X., Cao S., Cromie M., Shen Y., Feng Y., Yang H., Li L. (2017). Chlorogenic acid ameliorates experimental colitis by promoting growth of Akkermansia in mice. Nutrients.

[119] Hou J., Hu M., Zhang L., Gao Y., Ma L., Yan X., Xu Q. (2020). Dietary taxifolin potently protects against dextran sulfate sodium-induced colitis via NF-κB signaling, enhancing intestinal barrier and modulating gut microbiota. Front. Immunol..

[120] Dong Y., Lei J., Zhang B. (2020). Dietary quercetin alleviated DSS-induced colitis in mice through several possible pathways by transcriptome analysis. Curr. Pharm. Biotechnol..

[121] Abdel-Raheem I.T. (2010). Gastroprotective effect of rutin against indomethacin-induced ulcers in rats. Basic Clin. Pharmacol. Toxicol..

[122] Zhang W., Han F., He J., Duan C. (2008). HPLC-DAD-ESI-MS/MS analysis and antioxidant activities of nonanthocyanin phenolics in mulberry (*Morus Alba* L.). J. Food Sci..

[123] Kim H., Chung M.S. (2018). Antiviral Activities of mulberry (*Morus Alba*) juice and seed against influenza viruses. Evid Based Complement. Alternat. Med..

[124] Wang Y., Shao S., Guo C., Zhang S., Li M., Ding K. (2020). The Homogenous polysaccharide SY01-23 purified from leaf of *Morus Alba* L. has bioactivity on human gut *Bacteroides ovatus* and *Bacteroides cellulosilyticus*. Int. J. Biol. Macromol..

[125] Chen T., Shi N., Afzali A. (2019). Chemopreventive effects of strawberry and black raspberry on colorectal cancer in inflammatory bowel disease. Nutrients.

[126] Huang K., Dong W., Liu W., Yan Y., Wan P., Peng Y., Xu Y., Zeng X., Cao Y. (2019). 2-O-β-D-Glucopyranosyl-L-ascorbic acid, an ascorbic acid derivative isolated from the fruits of *Lycium Barbarum* L., modulates gut microbiota and palliates colitis in dextran sodium sulfate-induced colitis in mice. J. Agric. Food Chem..

[127] Huang K., Yan Y., Chen D., Zhao Y., Dong W., Zeng X., Cao Y. (2020). Ascorbic acid derivative 2-O-β-D-Glucopyranosyl-L-ascorbic acid from the fruit of *Lycium Barbarum* modulates microbiota in the small intestine and colon and exerts an immunomodulatory effect on cyclophosphamide-treated BALB/c Mice. J. Agric. Food Chem..

[128] Jurenka J. (2008). Therapeutic applications of pomegranate (*Punica Granatum* L.): A review. Altern. Med. Rev..

[129] Shaygannia E., Bahmani M., Zamanzad B., Rafieian-Kopaei M. (2016). A review study on *Punica Granatum* L.. J. Evid. Based Complementary Altern. Med..

[130] Chan-Blanco Y., Vaillant F., Mercedes Perez A., Reynes M., Brillouet J.-M., Brat P. (2006). The noni fruit (*Morinda Citrifolia* L.): A review of agricultural research, nutritional and therapeutic properties. J. Food Compost. Anal..

[131] West B.J., Jensen C.J., Westendorf J., White L.D. (2006). A safety review of noni fruit juice. J. Food Sci..

[132] Potterat O., Hamburger M. (2007). *Morinda Citrifolia* (noni) fruit—Phytochemistry, pharmacology, safety. Planta Med..

[133] Graf D., Di Cagno R., Fåk F., Flint H.J., Nyman M., Saarela M., Watzl B. (2015). Contribution of diet to the composition of the human gut microbiota. Microb. Ecol. Health Dis..

[134] Cao Y., Liu H., Qin N., Ren X., Zhu B., Xia X. (2020). Impact of food additives on the composition and function of gut microbiota: A review. Trends Food Sci. Technol..

